# Ultrathin Submicrometer Scale Multicolor Detector of Visible Light Based on Metamaterial

**DOI:** 10.3390/s19194103

**Published:** 2019-09-23

**Authors:** Young Jin Lee, Youngsoo Kim, Seokhyeon Hong, Soon-Hong Kwon

**Affiliations:** Department of Physics, Chung-Ang University, Seoul 06974, Korea; youngjin.lee.91@gmail.com (Y.J.L.); youngsoo.kim94@gmail.com (Y.K.); lechter@naver.com (S.H.)

**Keywords:** surface plasmon, metamaterial, color detector

## Abstract

In this study, we propose a multi-color detector using a simple plasmonic metamaterial structure consisting of a silver and a indium phosphide. The color detector is composed of a metal strip with a periodicity in the *x*-axis direction on a layer of the dielectric material located on a metal substrate. This color detector can control the spectrum absorbed in the dielectric material layer by changing the thickness of the dielectric material layer or the width of the metal strip. The triangle formed by the three primary colors, namely, red, green, and blue, which are representatively detected by optimizing the color detector using only silver and indium phosphide, covers 44% of the standard Red Green Blue (sRGB) region. Furthermore, the area of the triangle obtained by further optimization, such as changing the material to gold or gallium phosphide or changing the period of the metal stirp, can aid in the detection of a larger number of colors covering 108% of the sRGB area.

## 1. Introduction

Metamaterials, which have been attracting increasing attention recently, exhibit a special phenomenon owing to their unique optical properties, which are not exhibited by natural materials [1,2,3]. Metamaterials have been representatively applied in the field of cloaking because they can alter the optical geometry as desired by controlling their permittivity and permeability [4,5,6]. In addition, metamaterials have also been applied to optical filters [7,8] and phasers [4,5,6,9], owing to their ability to control wavefront polarizations. Moreover, the strong light–matter interactions achieved in metamaterials enable an increase in the efficiency of ultrathin devices by confining the electric fields to the active layer of the device [10,11,12,13,14,15,16].

A color detector that detects the color of incident light is essential for creating a human-like optically constructed system [17], artificial retinas [18], and hyperspectral imaging [19,20]. In particular, a color detector in the visible light region is vital, because it can detect information in a similar manner to visual human perception. However, the majority of the color detectors that have been previously studied require materials with bandgaps matching the respective colors to detect multiple colors. The limitations arising from the use of multiple materials ultimately complicates the structure of multicolor detectors. In addition, hundreds of nanometer-thick color detectors are required to create artificial retinas.

In this study, we propose a metamaterial structure that strongly focuses the light of a specific wavelength on the absorption layer. Using this metamaterial structure, even when a single material with a fixed bandgap is used as the active layer, the detected color can be different, depending on the metamaterial structure parameters [21,22]. In this study, we simulated the proposed structures to detect the three primary colors, namely, red (R), green (G), and blue (B), by controlling the parameters of the metamaterial composed only of silver (Ag) and indium phosphide (InP). Applying the same principle as that of the human eye, the color detector is able to recognize various colors by combining the three primary colors already detected in the proposed metamaterial. In addition, we further optimized the three primary detected colors to appear more vivid by varying the metals, active material, and period of metamaterials, while maintaining the basic shape of the simple structure. The simple structure enables the development of extremely thin color detectors with a total physical thickness of less than 200 nm. Furthermore, the color detectors also have a high spatial resolution owing to their small pixel size. 

## 2. Materials and Methods

**Figure 1:**
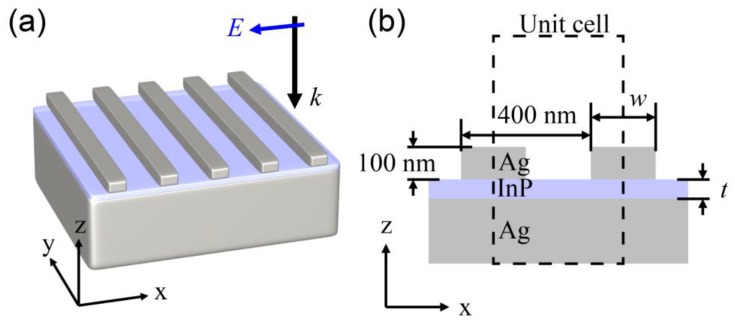
(**a**) 3D and (**b**) 2D schematics of the color detector. The Ag rectangular parallelepiped shaped strips are placed on the active layer. The strips are arranged with a one-dimensional periodicity of 400 nm and a height of 100 nm. The active layer composed of InP is between the Ag substrate and the strips. The width of each strip (*w*) and thickness of the layer (*t*) are key parameters to control the absorption of the active layer. The unit cell for computing is defined from the center of the strip to the center of the next strip.

We theoretically proposed a subwavelength thickness RGB color detector by using a metamaterial consisting of metal (Ag) strips on an ultrathin high-absorbing semiconductor material (InP) planar layer with a silver substrate, as shown in Figure 1. The silver strips with a width of *w* and a height of 100 nm were placed with a one-dimensional periodicity of 400 nm. The thickness of the InP layer is represented as *t*. The detected color can be easily controlled by adjusting the width of the silver strips and thickness of the InP layer. The color detectors have a limited operation spectral range, which determines the detection color. Here, the detection color was defined by converting the absorbance spectrum into the corresponding color. In addition, we further optimized the color detector to cover a broader color space by changing the metal to gold (Au) or the semiconductor layer to gallium phosphide (GaP) or by using a wide periodicity. The optical properties of the metals Ag and Au and dielectric material, InP, are applied to the simulations by referring to the papers written by Johnson [23] and by Aspnes [24], respectively. InP is also considered as dispersive media for the wavelength range from 400 nm to 700 nm.

The InP semiconductor layer was employed as an absorbing material for the detector with a varied thickness (*t*) less than 100 nm, which is much smaller than the target wavelength range of visible light. The silver strips with a height of 100 nm were placed on the InP layer with a one-dimensional periodicity (*a*) of 400 nm along the *x*-axis. In addition to exciting the resonance in the InP layer for the incident light, the silver strip and substrate were also employed as electrodes to receive the carriers generated by the incident light. The InP layer can absorb only light of a desired color when the appropriate structure parameters, *w* and *t*, are chosen. The absorbed spectrum can also be controlled by changing the periodicity and height of the strips or by using another metal or absorber. The effective detector thickness estimated as the sum of the thickness of the InP layer and height of the silver strips was less than only 200 nm. 

We determined the absorption spectrum of the InP layer by using the finite element method (COMSOL Multiphysics [25]). Absorption spectrum with different values depending on the wavelengths makes human feel the colors by stimulating the three cone cells of the human eye differently. The detection process can be similarly realized using a Commission Internationale de l’Eclairage (CIE) Coordinate Calculator [26]. Then, the absorption spectrum was converted to the detection color by using the CIE Coordinate Calculator [ref]. We chose three structures that could measure the red, blue, and green detection colors to form the largest triangle in the CIE color space. By using the detected values in the three RGB detectors, one can determine the color of the incident light, provided that the color is placed inside the triangle with three points, indicating the corresponding RGB colors, in the CIE space. 

In COMSOL Multiphysics, the boundary condition on both sides of the unit cell was applied with a periodic boundary condition for achieving same environment in which metal strips are arranged periodically along the *x*-axis. The upper and lower sides of the unit cell was set to the ports. The normal incident light is assumed by setting the upper port to excite the incident wave. We measured the absorption of the active layer by detecting reflection and transmission at the upper and lower ports while the wavelength of the incident light changes from 400 to 700 nm. In this finite element method (FEM), we measured the absorption of the active layer by detecting reflection and transmission at the upper and lower ports. In addition, the absorption was also calculated by spatially integrating resistive loss, the inner product of the current density and electric field, over the active layer. Both methods match within the error tolerance.

## 3. Results and Discussion

**Figure 2:**
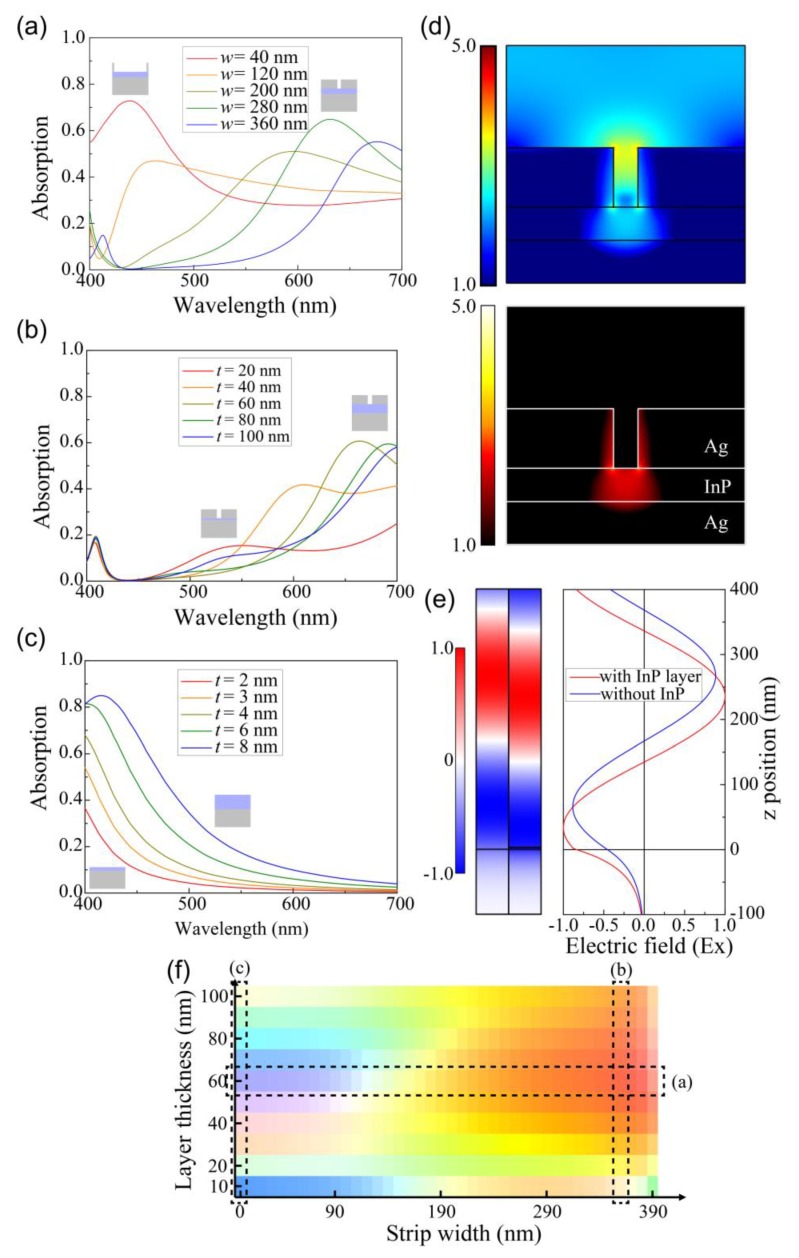
Absorption spectrums detected by the metamaterial detectors (**a**) when the width (*w*) of the strip varies with the thickness (*t*) of the indium phosphide (InP) layer fixed at 60 nm; (**b**) when *t* varies with *w* fixed at 350 nm; and (**c**) when *t* varies without the Ag strips. The insets depict the narrowest/widest width (*w*) and thinnest/thickest thickness (*t*) structures. (**d**) Electric field intensity (up) and absorption mode profiles (down) when *w* = 359 and *t* = 56 nm, in which the color red is mostly detected. (**e**) Field profiles of the x component of the electric field (*E_x_*) when light is incident on an Ag substrate with or without a 3-nm InP layer for the incident light with a wavelength of 400 nm. The left side profile and red line represent the *E_x_* field when the light is incident on the Ag substrate. The right side profile and blue line represent the *E_x_* field with a 3-nm InP layer on the same substrate. (**f**) Detected color by the color detector for various strip widths and InP layer thicknesses.

In our simulation, we investigated the absorption spectrum as functions of the width (*w*) of the Ag strip and thickness (*t*) of the InP layer. Figure 2a–c illustrate the absorption spectrums as a function of each parameter, such as *w* with *t* fixed at 60 nm (Figure 2a); *t* with *w* fixed at 350 nm (Figure 2b); and *t* without Ag strips on the InP layer (Figure 2c). The absorption spectrum changed when *w* or *t* changed, because the plasmonic resonance that corresponds to the peak of the absorption spectrum was excited at the edge of the strips, and the absorption spectrum’s wavelength depends on *w* and *t* [10,15]. The wider *w* in Figure 2a moved the absorption spectrum’s peak to the longer wavelength region, because the resonant wavelength of the metal–insulator–metal (MIM) mode generated in the gap between the Ag strips shifted to the red region when the gap decreased due to the larger effective refractive index [27]. The thicker *t* in Figure 2b also moved the peak to the longer wavelength region, because a larger field overlap of the plasmonic mode in the InP layer induces a larger effective index. Figure 2d depicts the electric field intensity and absorption mode profile in a structure with Ag strips for the plasmonic mode with the peak wavelength of the absorption spectrum. Light is strongly absorbed in the corners of the Ag strips and the InP layer just under the air gap, where the electric field intensity is strongly confined.

Furthermore, the absorption can be significantly enhanced by the interference effect [28] owing to the strong phase change in the large absorption coefficient layer in the ultrathin layer, although the layer is too thin to generate the conventional Fabry–Perot resonance. Even with an 8-nm thickness of the InP layer, the absorption peak at 415 nm reached 85%. The absorption peak also shifted to the longer wavelength region for the thicker InP layer, as depicted in Figure 2c. To understand this large absorption phenomena, we investigated the reflected phase change of the thin InP layer (*t* = 3 nm) in comparison with the case with the silver substrate without the InP layer. 

Figure 2e depicts the x component of the electric field (*E_x_*) with and without the 3-nm InP layer on the Ag substrate. The maximum point of *E_x_* moved by 33 nm from *z* = 235 to 268 nm due to the existence of the 3-nm thick InP layer, which is extremely small when compared with the light wavelength of 400 nm. The ultrathin InP layer placed on the flat silver is suitable for detecting blue color light, because strong resonant absorption is observed when an extremely thin highly-absorbing media is placed on the metal with a finite optical conductivity [28].

Based on the absorption spectrum for various strip widths and thicknesses of the InP layer, Figure 2f illustrates the color absorbed in the proposed detector structures. Here, the detected color was obtained by converting the absorption spectrum into the color using the CIE Coordinate Calculator [26]. Therefore, we can control the detected colors (blue, green, and red) as required, as depicted in Figure 2f, because the spectrum can be varied by varying *t* and *w*. The left side of Figure 2f depicts the detected color when *w* is 0 nm, i.e., there is no Ag strip on the InP layer, and the structure consists of only the InP layer and the Ag substrate. As depicted in Figure 2f, the blue color can be detected by the structure when *w* = 0 nm and the values of *t* is less than 10 nm. 

**Figure 3:**
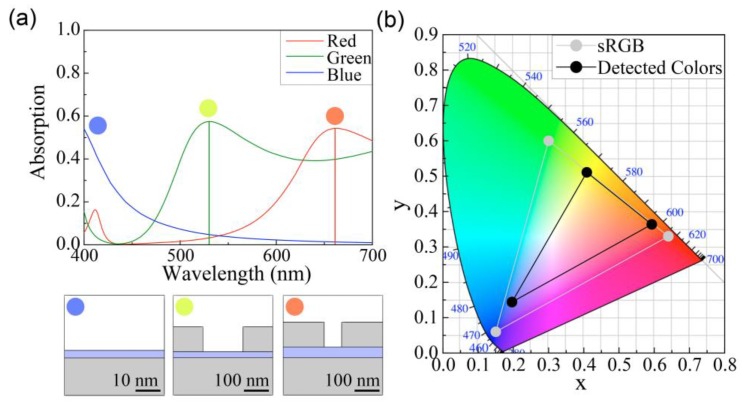
(**a**) Absorption spectrums and (**b**) color coordinates in the CIE color space for three representative detector structures for the colors red, green, and blue. The red and green colors are detected by the structure in which the Ag strips have values of width (*w*) = 357 nm, thickness (*t*) = 56 nm, and *w* = 239 nm, *t* = 24 nm, respectively. The blue color is detected by the structure of the InP layer with *t* = 3 nm on the Ag substrate without the Ag strips. The scaled schematic of each structure is depicted below the graph. The colors detected by each structure are plotted as the colored solid circles, as depicted in the insets of (**a**). The triangular region between the three black dots in the CIE color space in (**b**) represents the possibly detectable colors for the proposed color detectors. This region covers 44% of the sRGB (gray).

Figure 3a illustrates the absorption spectrum of the three structures to detect the red, green, and blue colors. The colors in the circles in Figure 3a were obtained from the chromaticity values determined by transforming each absorption spectrum. In the structure with *t* = 56 and *w* = 357 nm, the resonant peak of the red color spectrum was maximum at λ = 662 nm. The peak of the green color spectrum was maximum at λ = 531 nm in the structure with *t* = 24 and *w* = 239 nm. To detect the blue color, a thin InP layer with a thickness of 3 nm on the Ag substrate was used without any Ag strip (*w* = 0 nm). The peak absorbance of each structure was larger than 50% despite the deep subwavelength thickness of the absorption layer that composed InP, which was less than 100 nm. In the structure with metal, there were two absorptions: the metallic absorption and the InP absorption. In this paper, we consider only the absorption in the InP active layer in the absorption spectrum, which can contribute to the operation of photodiodes. The corresponding colors of the absorption spectrum in Figure 3a are represented in the CIE color space, as shown in Figure 3b. The three color points (black dots) were chosen to have the largest triangle area while maintaining the proposed structure consisting of the InP layer and Ag strips and substrate. The chromaticity values of the detected color of the proposed structures (black) and standard red green blue (sRGB) (gray) are depicted in Figure 3b. The triangular region of the optimized color detector, which represents the possible colors that can be detected, covered 44% of the sRGB color region. In other words, we can distinguish 44% of the colors that are represented by an sRGB-based device. The insets of Figure 3a, which indicate the detected colors, show the relatively low-chromaticity colors: namely, red, green, and blue.

**Figure 4:**
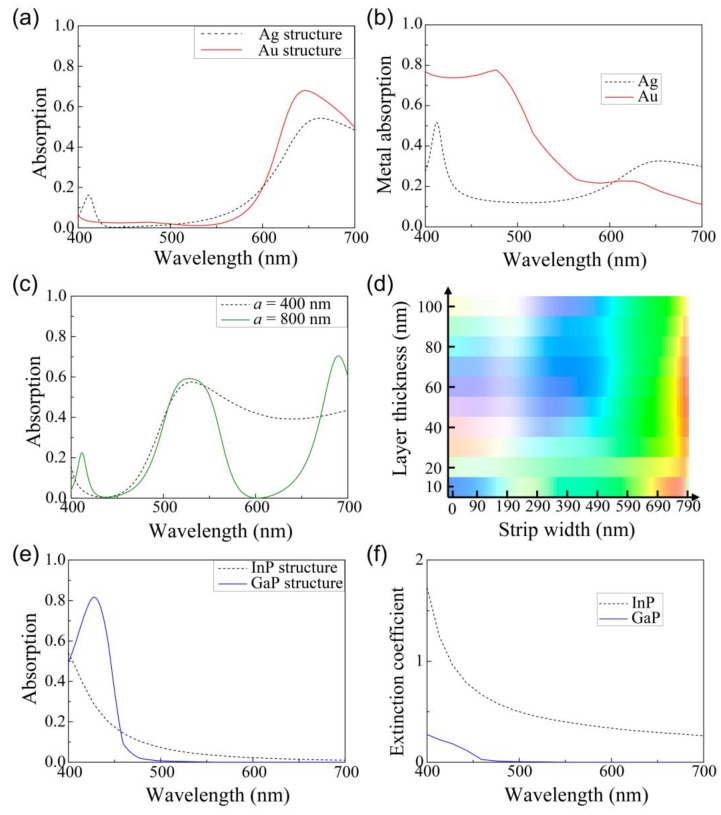
Absorption spectrums of the absorption semiconductor layer in the three representative structures (black dotted lines) of Figure 3 and the three additionally optimized structures (red, green, and blue solid lines) for the colors of (**a**) red, (**c**) green, and (**e**) blue. The red optimized structure has the structure parameters of *w* = 331 nm and *t* = 44 nm, in which Ag is replaced by Au. The green structure (*w* = 669 nm, *t* = 96 nm) has a different period of the Ag strips (*a’* = 800 nm), and the blue structure (*t* = 64 nm) has a gallium phosphide (GaP) layer as the absorption layer instead of the InP layer. (**b**) Absorption spectrums of the metals, Ag (black dotted line) and Au (red line), in the structures detecting the red color. (**d**) Colors detected by the structure with a double period *a’* of 800 nm for various strip widths and InP layer thicknesses. (**f**) Imaginary part of the refractive index for the absorption layers, InP (black dotted line) and GaP (blue line).

In Figure 3, we suggested three representative structures to detect the three colors—namely, red, green, and blue—by varying the thickness (*t*) of the InP layer and the width (*w*) of the Ag strip to obtain the largest detectable color area in the CIE color space, while maintaining the material compositions (InP, Ag) and the period of the strips (400 nm). To achieve a higher chromaticity detected color, we investigated the further optimization of the three colored structures by varying the metal (Figure 4a,b), period of the strip (Figure 4c,d), and absorption material (Figure 4e,f). In each additionally optimized structure, the thickness (*t*) of the absorption layer and the width (*w*) of the metal strip were scanned for the further optimization of the detected colors.

First, the red color structure was re-optimized by changing the metal from Ag to Au. The broad peak in the short wavelength region of 400–500 nm in the absorption spectrum (black dotted line) of the InP layer of the structure with Ag was suppressed in the case (red line) of the structure with Au, as depicted in Figure 4a. This is because Au absorbs significantly more light than Ag in the wavelength range of 400–500 nm, as illustrated in the absorption spectrum of the metals including the strips and substrate in Figure 4b. Additionally, the InP absorption was further enhanced in the long wavelength region of 600–700 nm due to the lower absorption of Au than that of Ag. Consequently, the InP absorption spectrum of the optimized structure with *w* = 331 nm and *t* = 44 nm exhibited a more vivid red color.

Similarly, the blue color structure was re-optimized by changing the absorber from InP to GaP, which suppressed the absorption in the long wavelength region. The absorption spectrum (black dotted line) of the InP layer in the structure detecting the blue color in Figure 3 shows the non-negligible absorption in the long wavelength region in Figure 4e. On the other hand, when the InP layer was replaced with a GaP layer, the absorption (blue line) in the long wavelength region was strongly suppressed, owing to the larger absorption at 428 nm. This is because the extinction coefficient of GaP has a meaningful value only below a wavelength of 460 nm, whereas that of the InP has a considerably large value over the whole visible wavelength range. 

The green color structure was optimized in a different way. In the structures depicted in Figure 3, *w* was limited to a value up to 400 nm, because *w* must be smaller than *a*, which was fixed at 400 nm. Due to this limit, the near-infrared peak cannot be removed by choosing any *t* and *w*. For further optimization of the green color, the period was increased to 800 nm, so that a larger range of width could be chosen. In contrast to the structure with *a* = 400 nm, where the green color was rarely observed, in the structure with *a* = 800 nm, the green color was more vivid and more commonly detected by the structure with a wider *w* of approximately 600 nm, as illustrated in Figure 4d. 

**Figure 5:**
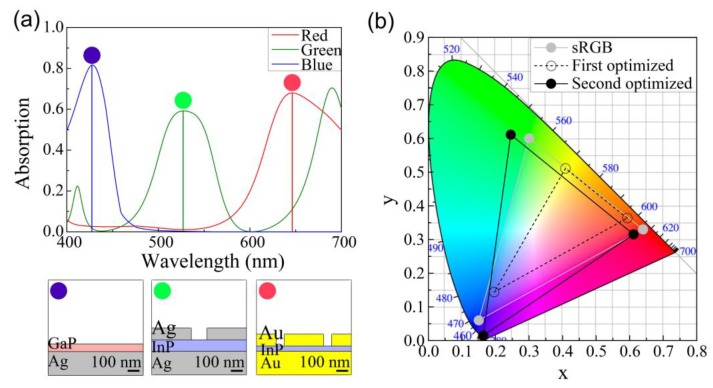
(**a**) Absorption spectrums and (**b**) color coordinates in the CIE space for the second optimized structures. The red, green, and blue colors are detected by each structure depicted in Figure 4a,c,e, respectively. The colored circles indicate the detected colors corresponding to the absorption spectrum. First (open circle, dashed line) and second (solid circle, solid line) optimized structures of (**b**) represent the structures in Figure 3 and Figure 4, respectively.

Figure 5a illustrates the absorption spectrum for the red, green, and blue colors for the second optimized structures depicted in Figure 4a,c,e. The colors detected by the second optimized structure were more vivid when compared to those detected by the first optimized structure in Figure 3, which can be clearly observed in the converted colored circles of Figure 3 and Figure 5. Figure 5b depicts the color coordinates of (a) in the CIE color space. The first optimized structure covers only 44% of the sRGB region by changing only *t* and *w*. The second optimized structure detects a larger number of colors with a larger gamut in the triangle, as depicted in Figure 5b, in which the area is even larger than the sRGB (gray line) region, i.e., 108% of the sRGB region. 

## 4. Conclusions

We proposed a color detector that uses plasmon resonance to efficiently detect visible light. The color detector, consisting of a layer of dielectric material sandwiched between the metal substrate and the periodic metal strips, absorbs lights with a wavelength-dependent efficiency. The absorption spectrum calculated by COMSOL Multiphysics [25] can be converted into the detectable colors by using the CIE Coordinate Calculator [26]. When selecting the three primary colors, namely, red, green, and blue, we chose to have the largest area in the CIE color space because the colors inside the triangle were detectable using a combination of the three color detectors, similar to the perception of the color of an object by a human.

In the first optimized structure composed of only Ag as the metal and the InP as a dielectric material, the width of the metal strip and the thickness of the InP layer were changed to optimize the detected colors with a larger gamut. In structures that detected colors corresponding to red (*w* = 357, *t* = 56) and green (*w* = 239, *t* = 24), the peak of the absorption spectrum could be controlled depending on *w* and *t* due to the MIM plasmon resonance formed between the strips. The detector for the blue color strongly absorbed the light in a thin InP layer of only 3-nm thickness without the Ag strips. Consequently, 44% of the sRGB region could be detected by the first optimized structure.

In the second optimized structure, the red color was further optimized by changing the metal from Ag to Au, because Au absorbs more light than Ag in the short wavelength region near 415 nm, and the net absorption of the InP layer decreases in the short wavelength region. On the other hand, the blue color was optimized by changing the dielectric material from InP to GaP, because GaP has a negligible extinction coefficient at long wavelengths. To optimize the green color, we changed the periodicity of *a* = 400 nm to *a* = 800 nm, which increased the possible range of the strip width without changing the metal or dielectric material. The region of the detectable colors in the second optimized structures was 108% of the sRGB region in the CIE color space. In other words, the sRGB-level colored image could be detected by the proposed color detectors if the set of color detectors, red, green, and blue, are arranged. The proposed detector has the thickness of only less than 200 nm, and the size of a single detector is smaller than 1 μm. Therefore, submicrometer, ultrathin color detectors with a larger color gamut based on the proposed structure can be exploited in numerous applications, such as artificial retina [18] and various color image sensors [19,20].

In the proposed structures, light intensity in the InP layer is strongly enhanced by exciting localized plasmonic modes at the corners of the Ag strips or inducing the interference effect [28] in the ultrathin, highly absorbing media, InP layer. The enhanced light intensity contributes to increase more photogenerated carriers, which can be detected as a photocurrent by using the silver strips and substrate as electrodes.

We demonstrated that various colors can be detected with a simple unit structure composed of a solitary metal and a dielectric material, by controlling the width of the metal strip and thickness of the layer. Therefore, the detectors can be applied to multiple devices that detect specific wavelengths. In addition, the performance of the color detectors can be improved by selecting other materials or changing the parameters that have not been considered in this study.
